# Morphological Buffering in Neuro-Architecture: Interactive Effects of Color, Shape, and Area Proportion on Affective Processing

**DOI:** 10.3390/bs16081364

**Published:** 2026-08-09

**Authors:** Xiaoxiao Dou, Yan Zhang, Yannan Zhang, Mengyao Kang, Qiangqiang Fan, Xiaona Xie, Yufan Sun, Mengyao Li

**Affiliations:** College of Arts, Northeastern University, Shenyang 110819, China

**Keywords:** neuro-architecture, electroencephalography (EEG), virtual reality (VR), dual-system processing, morphological buffering

## Abstract

While architectural visual features significantly influence human emotion, the spatiotemporal neuro-mechanisms underlying their interactive effects remain poorly under-researched. This study systematically decoupled the dynamics of indoor spatial perception by investigating the interactive impacts of color combination, color shape, and area proportion to find out the effects on participants’ emotion. By integrating high-resolution electroencephalography (EEG), specifically analyzing the N200, late positive potential (LPP), Frontal Alpha Asymmetry (FAA), and Beta bands with Self-Assessment Manikin (SAM) ratings and Liking scores in immersive virtual indoor environments, data from 61 valid participants were analyzed. The results revealed a hierarchical neuro-affective processing structure: morphology dominated early cognitive processing, where curved geometries acted as a pre-attentive cognitive buffer, acting as a cognitive buffer by significantly attenuating the early-stage visual stress induced by angular, high-contrast configurations. Conversely, color combination primarily drove late-stage sustained environmental arousal, tracked by the LPP. Notably, area proportion demonstrated a significant amplification effect, significantly exacerbating pre-existing neuro-affective biases. Crucially, cross-modal triangulation revealed a selective inter-modal coupling: while sustained cortical activation (ΔLPP) significantly predicted subjective emotional arousal, early physiological buffering (ΔN200/ΔFAA) was functionally dissociated from subjective aesthetic preference (ΔLiking). This decoupling provides preliminary evidence consistent with a dual-system processing framework, suggesting that while curves may facilitate a preconscious biological safe baseline, ultimate aesthetic appreciation is likely subject to top-down cognitive over-riding. Ultimately, these findings transition indoor spatial color design from intuitive practice to evidence-based neuro-aesthetics, highlighting the critical role of morphological buffering and EEG metrics in optimizing human emotional well-being.

## 1. Introduction

The relationship between the inner space of a building and human perception is relatively complex, influenced by factors such as scale, light, color, and form, etc. (Vartanian et al., 2015). Previous research shows that visual elements of an architectural environment not only shape the experience of subjects, but also regulate the activities of the autonomic and central nervous systems, thereby affecting people’s mental health and behavior (Bower et al., 2022). There has been some progress in some new interdisciplinary fields such as neuro-architecture. Recent advancements in this field suggest that these reactions are not only subjective preferences, but also a consequence of neurophysiological responses to spatial variables (Gregorians et al., 2026).

### 1.1. Key Visual Elements in a Space: Color Combination, Color Shape, and Area Proportion

Color combination, color shape, and area proportion are key indoor visual variables, which can arouse emotional reactions and psychological responses. These factors systematically modulate a bottom-up sensory process and a top-down spatial aesthetic evaluation. Studies have shown that color temperature and harmony determine the emotional valence of the space. Cool colors and analogous colors can arouse calm or relaxed emotions, while high-arousal complementary colors or warm colors can enhance physical activities. However, the precise emotional valence in a state of high arousal, whether it is positive excitement or negative discomfort, differs in various situations and cultural backgrounds (Jiang & Westland, 2024; Sun et al., 2024; Szabó et al., 2010). Other research shows that color parameters are directly adjusted from the autonomic nervous system (ANS); high-arousal visual stimuli trigger fast pre-attentive responses before conscious evaluation (Küller et al., 2009).

Beyond color, the form of spatial elements, especially angles and curves, is another key determinant in neuro-architecture. Evolutionary aesthetics holds that humans generally prefer curvilinear or streamlined profiles. This preference is deeply rooted in the brain’s reward circuit. Smooth visual form transitions are processed here into greater perceptual fluency and with less energy consumption (Gómez-Puerto et al., 2016). Such geometric shapes help to approach and produce positive emotions. Conversely, geometric shapes with sharp angles are often regarded as threatening and can trigger more intense activation of the amygdala.

The psychological impact of visual features in the physical environment is not static. The spatial range or color area is the quantitative amplitude of emotions (Alqamadi, 2025; Özler et al., 2023; Qiao et al., 2021). The key point is that the existing research mainly focuses on the steady state psychological output of isolated features (Jin et al., 2022), and there is a gap in the interactive regulation of the early cognitive processing of the brain in terms of color combination, color shape, and area proportion (Chen et al., 2023). Recent research shows that spatial perception is cross-modal; color preferences change with changes to different geometric shapes or patterns (Ulusoy, 2024; Vartanian et al., 2013; Zejnilovic et al., 2023). It is not yet clear whether a particular morphological structure can alleviate the negative emotional load caused by radical color scale combinations during the pre-attentive stage. Therefore, it is necessary to separate the basic perception effects from the semantic clutter factors of specific functional rooms, and explore the interaction of three factors in a controlled virtual box-type space, to be able to provide objective parameters to extract pure neurocognitive results.

### 1.2. Neurophysiological Markers: From Subjective Appraisal to Brain Dynamics

To evaluate the psychological impact of spatial features, traditional empirical research often uses self-report methods. For example, the SAM (Self-Assessment Manikin) (Bradley & Lang, 1994) and semantic differential scales are used to quantify the emotional responses of subjects regarding color combinations (Valdez & Mehrabian, 1994) and color shape (Vartanian et al., 2013). Self-report can provide insights into perceived comfort and the like, but it is easily affected by subject cognitive biases and the like (Podsakoff et al., 2003). What is important is that the questionnaire omits the instantaneous, unconscious emotional processes and the continuous neurophysiological fluctuations when people enter the space.

To break through the limitations, environmental psychology research has switched to using EEG to directly measure neurophysiological activities. Emerging empirical studies use EEG to decode spatial variables. For example, Llinares et al. (2021) combined psychological tests with EEG, showing that cold or warm indoor color schemes directly regulate neurophysiological responses, revealing the subconscious cognitive dynamics that traditional methods struggle to fully capture. Similarly, Banaei et al. (2017) used a computer to display the shape of the internal arch, showing curved and pointed contours, which affected the dynamics of the brain, in terms of emotional arousal and sensorimotor integration. Based on this empirical evidence, this study selects biomarkers from electroencephalography (EEG) and event-related potentials (ERPs) to capture the dynamics of different stages of the cognitive emotional process. In the frequency domain, the Beta band in the occipital–parietal region, with a range of 13 to 30 Hz, is monitored as a reliable indicator of general visual stress and active cortical arousal (Kim & Kim, 2025). Frontal Alpha Asymmetry (FAA) can accurately decode approach–avoidance trends and emotional values, and greater left prefrontal activation implies positive emotions (Harmon-Jones et al., 2010). In the time domain, the N200 event-related potential component mostly comes from the anterior cingulate cortex (ACC), reflecting cognitive conflict and the mismatch negativity; a larger N200 amplitude indicates that the brain spends more energy when processing visual inconsistencies or environmental threats such as acute angles in high-contrast spaces (Folstein & Van Petten, 2008). The late positive potential (LPP) is a reliable marker of sustained emotional arousal and motivational attention allocation (Hajcak et al., 2010).

Neuro-architectural studies have confirmed that highly immersive VR environments can trigger physiological and psychological reactions similar to those in the physically real world environment, thereby ensuring strong ecological validity (Higuera-Trujillo et al., 2017).

### 1.3. Study Objectives and Hypotheses

An important unresolved problem in neuro-architecture is whether specific morphological features can interfere with the pressure amplification process caused by extreme colors and scales. The evidence in neuro-architecture shows that morphological features can regulate emotional influence and aesthetic judgment (Vartanian et al., 2013), but how specific shapes interact with spatial regions to relieve visual pressure has not been quantified. Neurophysiological studies confirm that high-arousal chromatic environments can significantly modulate autonomic nerve activity and electroencephalogram (EEG) responses, often inducing visual stress and cognitive overload when applied extensively (Monger et al., 2015; Roy et al., 2021; Wilms & Oberfeld, 2018), and negative effects rely on simulating environmental spatial covariance and scale amplification (Stamps, 2010). A large-scale high-arousal color environment will inevitably produce serious psychological loads. Then, can the curve shape be regarded as a pretentious cognitive buffer that may reduce amygdala threat activity (Bar & Neta, 2007) and promote interpersonal space to be beneficial to brain dynamics (Banaei et al., 2017)? The potential point where this optimal morphological feature offsets the negative emotions of strong color scale is consistent with the emerging cognitive emotional framework of architectural design (Higuera-Trujillo et al., 2021), which is referred to as an “Emotional buffering effect” in this study to describe the observed attenuation of early visual stress, rather than to propose a new neurophysiological mechanism.

To solve the problems mentioned above, this research is designed to explore a question at the intersection of architectural design, neuroscience, and environmental psychology. The objective neurodynamic including EEG and ERP (N200, LPP, FAA, Beta) and the subjective psychological evaluation including relevant contents of SAM (Pleasure, Arousal, Dominance, and Liking) are combined, aiming to enhance the understanding of how visual variables layer-by-layer adjust physiological relaxation and emotional perception.

Based on the theoretical frameworks discussed above, this study aims to move beyond exploratory questions and test the following specific hypotheses (H):

**H1** **(Independent Effects).** *Specific visual elements can independently regulate different time stages of spatial perception. Specifically, the curve shape will reduce the processing process of early cognitive conflicts, the contrasting color combination mainly affects the late continuous awakening state, and the larger area proportion will amplify the overall emotional response*.

**H2** **(Two-way Morphological Buffering).** *The curved form acts as a kind of pre-attention cognitive buffer, interacting with color combination, and significantly alleviating the early visual pressure caused by high-contrast colors*.

**H3** **(Three-way Quantitative Amplification).** *The area proportion acts as a quantitative amplifier in the three-way interaction. It significantly intensifies the negative neuro-emotional response caused by angle and high-contrast configuration, while highlighting the necessity for the curve to exert a morphological buffer effect on an extreme spatial scale*.

## 2. Methodology

**Figure 1:**
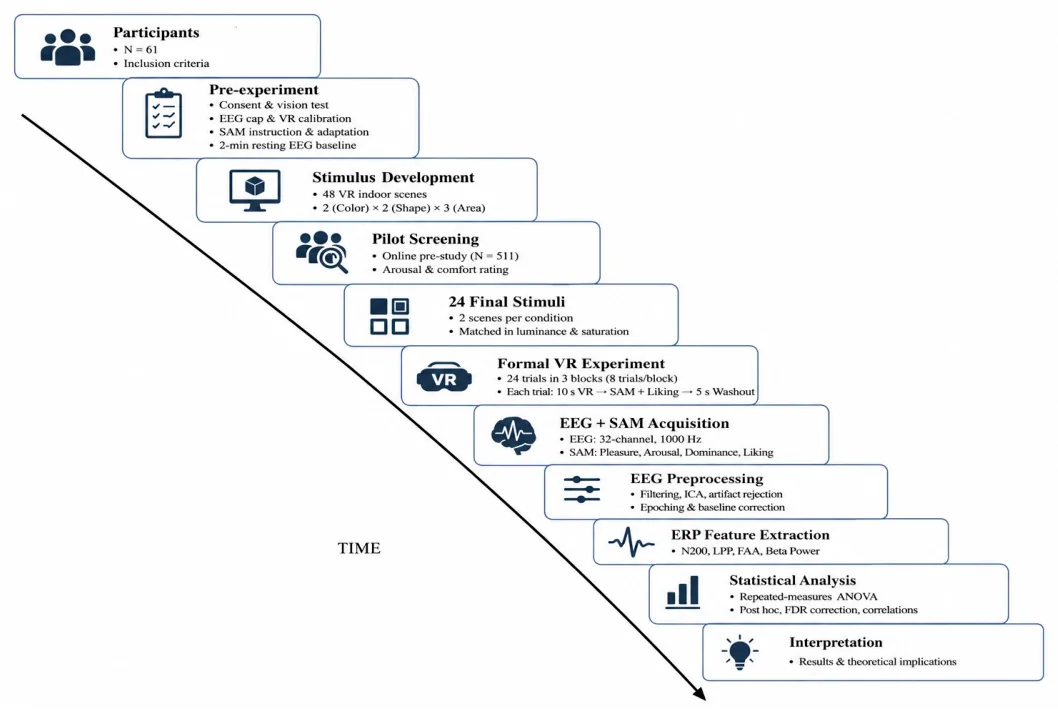
Research workflow and experimental design.

This study adopts a systematic multi-stage research process to explore the neuro-emotional processing process of indoor space variables. Figure 1 comprehensively outlines the research design and shows in detail the process from participant screening and stimulation development to the formal virtual reality electroencephalogram experiment stage, data collection, and subsequent analysis process.

### 2.1. Participants

A total of 63 participants were initially recruited for this study. Due to interference signals and poor EEG signal quality, the data of two participants were excluded. Therefore, the final valid sample included 61 participants (M = 22.56). All participants were right-handed and had normal vision or normal corrected vision. Before the beginning of the experiment, the Ishihara Color Vision Test (Marey et al., 2015) was used to strictly verify the normal color perception of the subjects to exclude any color blindness or color perception defects. In addition, the pre-screening questionnaire confirmed that all subjects had no history of neurological, mental or cardiovascular diseases. The subjects were required to avoid alcohol, caffeine and any drugs that affect the central nervous system for at least 24 h before the start of the experiment. After fully explaining the experimental process and safety procedures to all subjects, a written informed consent was obtained. This study strictly follows the ethical principles of the Declaration of Helsinki and has been officially approved by the Ethics Committee of Northeastern University, China (Approval No. NEU-EC-2025B054S). Each participant received financial compensation following the experiment. An a priori power analysis using G*Power 3.1 was conducted for a repeated measures ANOVA to determine the required sample size. To detect a specific targeted three-way interaction effect (Colo × Shape × Area, 12 measurements) with a medium effect size of *f* = 0.25, an alpha level of α = 0.05, and a statistical power of 0.80, the analysis assumed a correlation among repeated measures of 0.5 and a conservative nonsphericity correction (ε) of 1.0. The calculation indicated a minimum total sample size of *N* = 28. Therefore, the final valid sample size (*N* = 61) exceeded this requirement and provided sufficient statistical power to detect the hypothesized interaction effects.

### 2.2. Stimuli and Experimental Design

The study used a strict within-subjects experimental design to measure the interactive effects of physical space parameters, and the independent variables include three core dimensions:

Color combination: There were two effect levels in color matching. Similar colors like blue–purple and red–orange; complementary colors like red–green and yellow–purple (derived from Itten’s color theory) (Itten, 1970).

Color shape: Divided into two morphological levels. Angular: with sharp angles and square geometric figures. Curved: with circular and streamlined figures.

Area proportion: The area proportion refers to the percentage of the virtual field of view (FOV) covered by the target shape and color configuration. Specifically, these proportions are quantified by the coverage rate of the color internal surface in the central visual field of the participants. The proportion of 25% represents local decorative embellishment, 50% represents the balanced distribution of color and shape on the wall surface, and 100% corresponds to the complete immersive experience of the whole wall.

**Figure 2:**
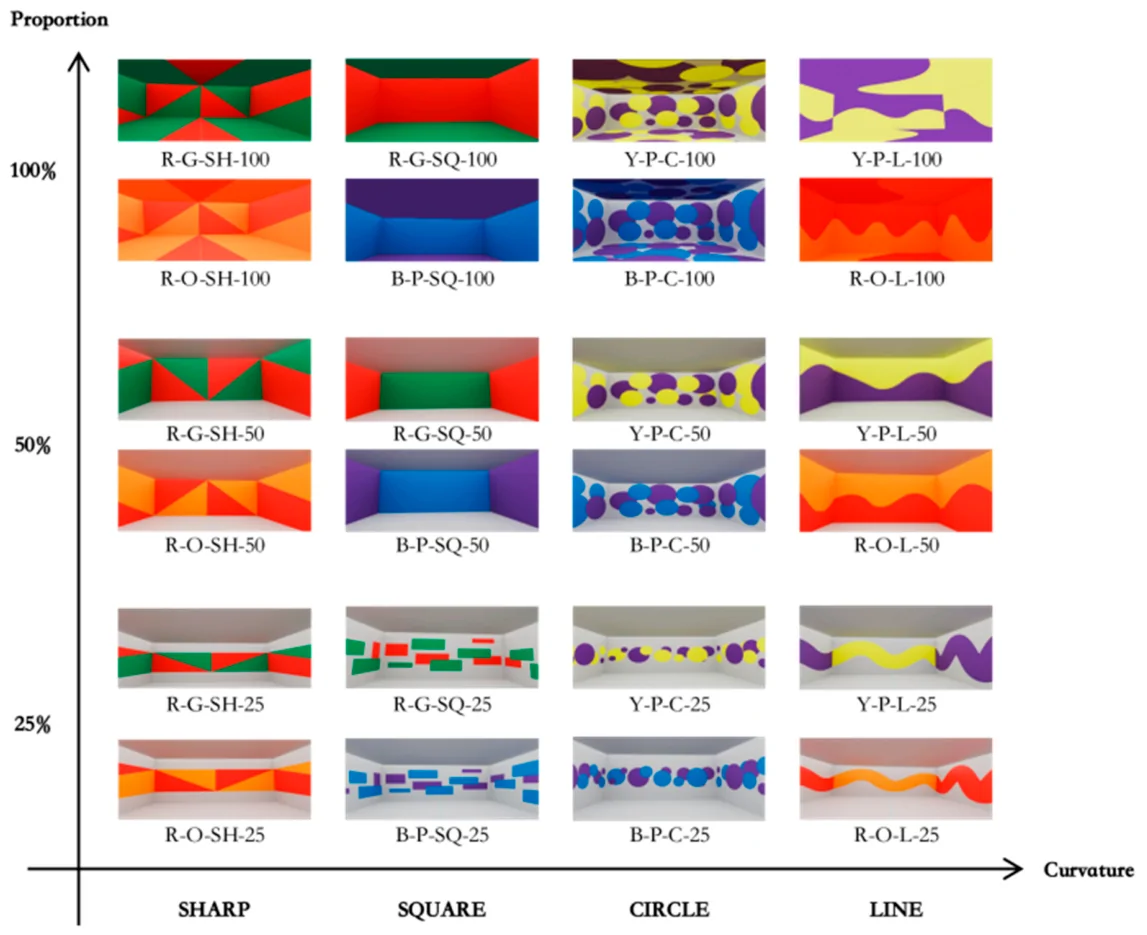
Examples of visual environmental stimuli: indoor scenes with different color combinations, contour shapes, and area proportions.

In order to ensure ecological validity and avoid single-stimulus bias, this study first constructed 48 color architectural space renderings through the combination of different physical elements. These initial stimuli were jointly designed and completed by 10 experts in the field of interior design and architectural psychology. In order to strictly screen the final experimental stimulus, the study further carried out a large-scale online pre-experiment (*N* = 511). Participants used the 7-point Likert scale to evaluate the emotional awakening and visual comfort of 48 architectural space renderings. The inter-rater reliability of the pilot evaluation was assessed using the intraclass correlation coefficient (ICC). The results showed good agreement among the participants for both emotional arousal (ICC(2, *k*) = 0.82) and visual comfort (ICC(2, *k*) = 0.79), indicating that the evaluations of the VR scenes were sufficiently stable for subsequent stimulus selection. To address the selection thresholds and ranking rules, a systematic screening procedure was implemented. First, to avoid serious VR dizziness caused by excessive visual stimulation, an exclusion threshold was set based on visual comfort: only stimuli maintaining acceptable visual comfort (mean comfort score ≥ 4.5) were retained. Second, among the retained stimuli within each spatial condition, scenes were ranked according to their mean emotional arousal ratings. The highest-ranked stimuli that best represented the intended perceptual manipulation of each experimental condition were selected. Following this systematic screening procedure, 24 VR scenes were retained as formal EEG experimental stimuli (Figure 2), so as to ensure that all main effects and two-factor and three-factor interaction effects could be estimated in the absence of statistical confusion. In order to reduce the sequence effect and fatigue effect, 24 VR scenes were presented in a balanced and random way using Latin design to ensure that any specific spatial conditions would not systematically appear at the beginning or end of the experiment, thus effectively reducing the sequence effect.

To ensure immersive realism and control confounding factors, 24 stimuli were modeled using 3D software and rendered in the Unity 3D engine to build a high fidelity VR environment (Higuera-Trujillo et al., 2017). To separate the effects of independent variables, irrelevant physical parameters such as basic illuminance, material reflectance, text roughness, and the participant’s VR view height were kept constant in all VR scenarios.

### 2.3. Measurements and Apparatus

The experiments were carried out in a strictly controlled laboratory with a temperature of about 23 °C to reduce external interference. To control additional variables, the spatial dimensions of all virtual stimuli were fixed at 4.0 m × 2.0 m × 6.0 m. Lighting used neutral white light (6500 K), color reproduction relied on the sRGB color space and was managed through a linear workflow to achieve precise chromaticity reproduction. In order to ensure that the perception difference is attributed only to the color, rather than mixing other visual variables, all stimuli were subjected to standardized chromaticity calibration. We specifically used a colorimeter to balance the average brightness (cd/m^2^) and saturation level under all conditions to ensure physical consistency. The specific RGB values (0–255) and HSV parameters of each color have been systematically listed in detail in the Supplementary material Table S1.

Participants experienced these immersive environments using an HTC Vive Pro headset; each eye had a high-fidelity resolution of 1440 × 1600 pixels and a refresh rate of 90 Hz, thus providing smooth and realistic visual stimuli (Le Chénéchal & Chatel-Goldman, 2018).

Continuous scalp electroencephalogram (EEG) signals were recorded by high-precision and wireless NeuroSci SDIAT-W32 electroencephalogram acquisition system (Lv et al., 2024). The device is equipped with 32 Ag/AgCl electrodes, whose location were arranged according to the international 10–20 system (Herbert, 1958). In addition, there are also two electro-ocular (EOG) channels specialized in monitoring eye movement (Jurcak et al., 2007). The system ensures high signal fidelity through Common Mode Rejection Ratio (CMRR) > 110 dB, input impedance ≥ 1 GΩ and input noise < 0.4 μVrms. The data were digitized at a sampling rate of 1000 Hz. In the preparation stage, conductive gel was applied to ensure that the electrode impedance is maintained below 5 kΩ (Picton et al., 2000). In order to ensure synchronization between behavioral visual stimulation and physiological response, event markers are directly transmitted from the Unity script to the EEG amplifier through a dedicated communication protocol (Tromp et al., 2018).

**Figure 3:**
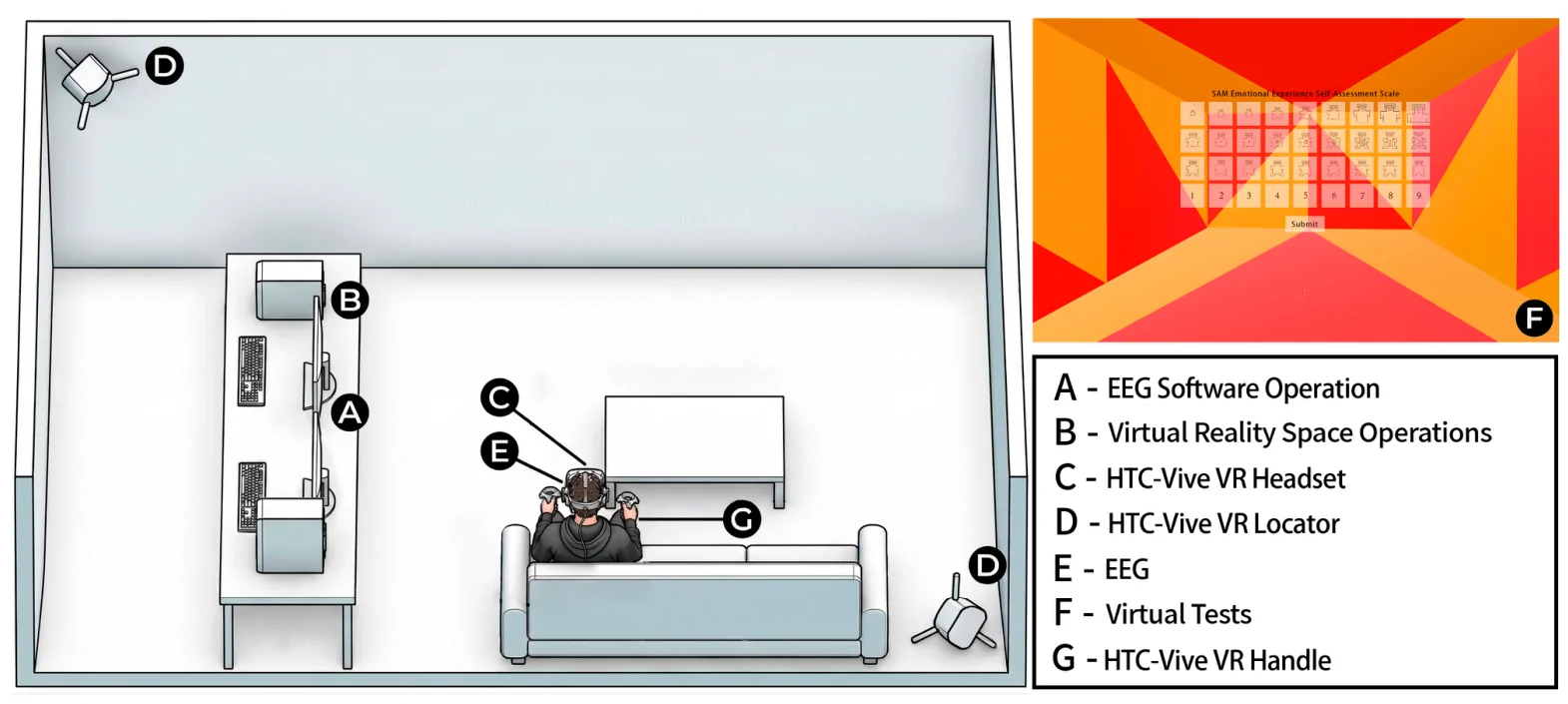
Schematic diagram of experimental setup and scene layout.

Immediately following each VR exposure, subjective emotional experiences were evaluated using the Self-Assessment Manikin (SAM) scale (Bradley & Lang, 1994). This internationally validated, non-verbal pictorial instrument employs a 9-point Likert-type design (Romeo & Testolin, 2025) to quantify three core affective dimensions: pleasure, arousal, and dominance. To maintain the immersion of the experiment and minimize the motor artifacts, the scoring interface was displayed directly in the VR headset, and the participants used the VR handle held in the right hand to record their answers. In addition, to evaluate the overall spatial aesthetic preference, a 9-point liking rating was added to this virtual interface. The integration of strict environmental control, high-resolution neurophysiological tracking, and standard subject assessment provides a reliable multidimensional framework for evaluating human–environment spatial interaction (Figure 3).

### 2.4. Experimental Procedure

Pre-experiment Phase: The subjects signed a written informed consent form and underwent the Ishihara color blindness test. Then, a 20 min preparation session was carried out, including putting on the EEG cap and using and calibrating the VR headset. Before the adaptation stage, the researchers explained the meaning of the SAM scale in detail to ensure that participants fully understood the scoring dimension. After that, subjects completed a brief virtual reality adaptation training to get familiar with the virtual environment and the evaluation procedure. Then, a 2 min EEG recording during an eyes-open resting state was performed to establish the physiological baseline.

**Figure 4:**
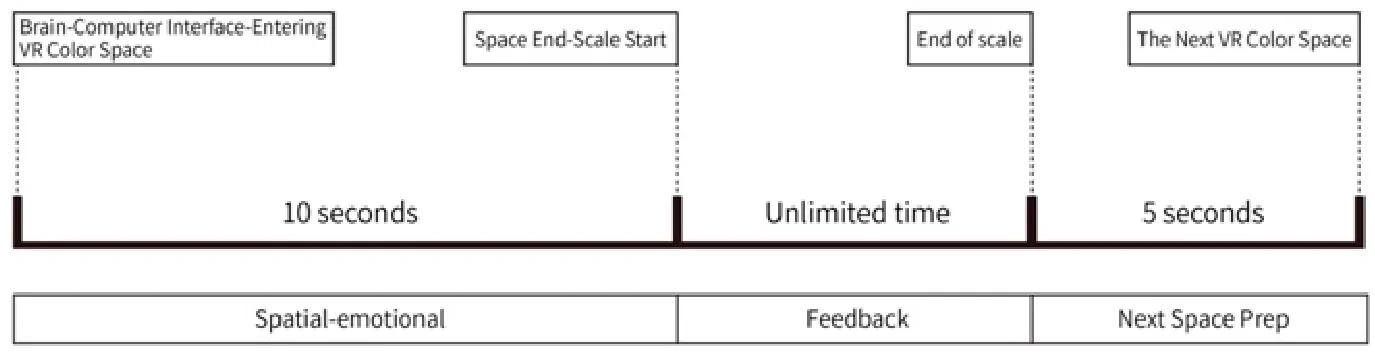
The timeline of a single trial experimental procedure.

Formal Experiment Phase: Participants sat in a comfortable position to view the virtual environment. The formal experiment contained 24 trials, which ware divided into 3 blocks, with 8 trials in each block. Between blocks, it provided a break to control the progress independently, during which they were allowed to temporarily take off the VR headset to relieve visual fatigue and prevent potential cybersickness. The presentation order of the 24 VR scenes was strictly balanced using a Latin square (Lewis, 1989), and the presentation or disappearance of stimuli were accurately synchronized between the Unity scripts and the EEG recording system (Coutray et al., 2025). Each trial proceeded in the following time sequence (Figure 4):

Stimulus Exposure Phase (10 s): Present the target virtual reality scene. Select 10 s as the time to obtain part of the immediate intuitive emotional response and the components of the continuous event-related potential, avoiding excessive cognitive rationalization (Kisker et al., 2021) or severe fatigue caused by VR exposure (Hajcak et al., 2010). During this stage, participants were instructed to blink as little as possible and move their bodies to reduce EOG artifacts, and avoid intentional emotional regulation.

Subjective Rating Phase: Following the stimulus, participants used the SAM scale and the Liking scale to score their emotional reaction. In this stage, they proceeded at their own pace and there was no time limit.

Washout Phase (Inter-Trial Interval, 5 s): A 5 s neutral pure white cubic space ended each trial, to eliminate the previous emotional residue through visual or psychological extinction, reset the visual field, and reduce fatigue before the next trial (Cha et al., 2020).

### 2.5. Data Processing and Statistical Analysis

The raw EEG data were processed offline using MATLAB (R2024a, MathWorks) and the open-source EEGLAB toolbox (v2024.0) to improve the signal-to-noise ratio (Delorme & Makeig, 2004). The preprocessing steps followed a standardized, rigorous sequence: (1) The EEG data were downsampled to 500 Hz while preserving the frequency range of interest and improving computational efficiency. (2) A 0.5–40 Hz band-pass filter was applied to remove slow drifts and high-frequency noise (Kappenman & Luck, 2010). (3) Bad channels were detected via visual inspection combined with automated EEGLAB algorithms and subsequently interpolated using the spherical spline interpolation method. (4) The data were re-referenced to the common average (Nunez & Srinivasan, 2006). (5) Independent Component Analysis (ICA) was performed using the automated ICLabel plugin. Artifactual components were automatically identified and rejected using a strict probability threshold of greater than 90% (Pion-Tonachini et al., 2019). For time-domain analysis, to ensure a robust signal-to-noise ratio for ERP analyses, the continuous EEG data recorded during the sustained exposure to each VR scene were segmented into epochs ranging from −200 ms to 1000 ms relative to stimulus onset. Baseline correction was subsequently performed using the pre-stimulus interval (−200 to 0 ms). To reduce the possible signal autocorrelation effect of overlapping time windows, this study adopted a sliding time window segmentation strategy with a controlled overlap ratio. This method has been widely verified in continuous emotional EEG research (Dmochowski et al., 2012), especially for continuous stimulation tasks that cannot clearly delimit the starting point of discrete stimuli. By calculating the autocorrelation function of each time window, the independence of the segmented data was tested. The results showed that the autocorrelation had decayed to an insignificant level within a sliding step, indicating that the adjacent time window still retained enough independent neural information. At the same time, the number of split time windows was controlled within the range of the number of attempts commonly used in ERP research to avoid artificially increasing the signal-to-noise ratio due to too many attempts. Therefore, the statistical effectiveness of this study mainly comes from the real individual differences among the participants, rather than the accumulation of the number of highly overlapping time windows. Subsequently, the baseline correction was carried out using the −100 ms to 0 ms pre-stimulus interval, and automatic artifact elimination was implemented. All time windows with voltage fluctuations exceeding ±100 μV were automatically deleted. In total, 24 VR scenes corresponded to 12 experimental conditions. Under the joint action of sliding segmentation and false artifact rejection, each participant retained no less than 30 valid time windows under each experimental condition. The average artifact rejection rate of all participants was 12.4% (SD = 3.2%), and each experimental condition retained an average of 42 valid time windows (range: 35–48). In addition, each participant had an average of 2.8 (SD = 0.9) ICA artifact components identified and eliminated. This data retention level is consistent with the conventional practices of current ERP research, which provided a reliable data basis for subsequent analysis. For frequency-domain indicators, the Welch power spectral density (PSD) estimation method was used to analyze longer periods (Welch, 1967).

Based on the grand average waveform and EEG hotspots, four neurophysiological indicators were extracted: (1) N200 amplitude: defined as the mean voltage within the 230–330 ms time window over the frontal–central cluster (Fz, F3, F4, FC1, and FC2) to evaluate early cognitive conflict detection. (2) LPP amplitude: quantified as the mean amplitude within the 400–800 ms window over the parietal–central region (Pz, CPz, Cz, P3, and P4) to reflect sustained emotional arousal and aesthetic evaluation (Weinberg et al., 2012). (3) Beta power: extracted from the 13–30 Hz frequency band and averaged across the occipital electrodes (O1, Oz, and O2) to evaluate visual stress induced by color stimulation (Wróbel, 2000). (4) Frontal Alpha Asymmetry (FAA): as an indicator of emotional value and approach–avoidance tendency, the calculation method was based on the natural logarithm of the difference between right and left frontal electrode power: *FAA* = *ln*(*Power F*4) − *ln*(*Power F*3), bandwidth frequency is 8–13 Hz (Davidson, 1998).

Statistical analysis was conducted using SPSS 27.0 (IBM). The data were integrated into a 2 × 2 × 3 repeated measures ANOVA (RM-ANOVA) framework for all subjective ratings and extracted neurophysiological indicators. Sphericity was evaluated using Mauchly’s test; if violated, degrees of freedom were adjusted via the Greenhouse–Geisser correction. The significance threshold was set at *p* < 0.05, and effect sizes were reported as partial eta-squared (η_p_^2^). To control for the increased Type I error risk associated with conducting multiple independent ANOVAs across diverse behavioral and EEG outcomes, we adopted the Benjamini–Hochberg False Discovery Rate (FDR) correction for the main effects and interactions. Each effect and the associated post hoc comparisons were strictly corrected by the Bonferroni method.

## 3. Results

### 3.1. Subjective Psychological Appraisals

Table 1 summarizes the descriptive statistics (mean ± SD) of the emotional dimensions (pleasure, arousal, dominance) of the subjects and the liking ratings under 24 experimental conditions. To systematically test the research hypotheses, a three-way repeated measures ANOVA (RM-ANOVA) was conducted, with the statistical results detailed in Table 2.

#### 3.1.1. Main Effects of Physical Spatial Parameters

Area proportion demonstrated the most extensive influence, showing a highly significant main effect in all dimensions: pleasure (F (1.53, 92.04) = 21.93, *p* < 0.001, η_p_^2^ = 0.268), arousal (F (1.42, 85.08) = 75.99, *p* < 0.001, η_p_^2^ = 0.559), dominance (F (1.40, 84.12) = 59.08, *p* < 0.001, η_p_^2^ = 0.496), and liking (F (1.45, 87.00) = 10.88, *p* < 0.001, η_p_^2^ = 0.158). Mauchly’s test of sphericity was conducted for all main effects and interactions involving the Area factor (which comprises three levels). The detailed results of the sphericity tests and the corresponding Greenhouse–Geisser correction estimates for all subjective measures are systematically reported in Supplementary Table S2. Where the assumption of sphericity was violated, degrees of freedom were corrected using Greenhouse–Geisser estimates. It is worth noting that as the spatial coverage increased from 25% to 100%, the arousal reported by the participants increased sharply (MD = 1.246, 95% CI [0.927, 1.564], *p* < 0.001). For example, under the extreme contrast–angular condition, the arousal increased significantly from M = 4.52 to M = 5.62 (MD = 1.10, *p* < 0.001, Cohen’s d = 0.76), and at the same time, the sense of dominance (from M = 5.67 to M = 4.03, M = 1.64, *p* < 0.001, Cohen’s d = 1.28) and the sense of pleasure (from M = 4.96 to M = 3.90, MD = 1.06, *p* < 0.001, Cohen’s d = 0.89) also decreased significantly. This shows that large-scale color stimulation will not only cause a strong sense of spatial oppression, but also produce significant negative emotions and visual fatigue (Stamps, 2011).

In addition, color shape has a significant main effect on spatial aesthetic preference and emotional control. Environments featuring curved contours yielded significantly higher pleasure (F (1, 60) = 27.62, *p* < 0.001, η_p_^2^ = 0.315), dominance (F (1, 60) = 7.62, *p* < 0.01, η_p_^2^ = 0.113), and liking scores (F (1, 60) = 20.58, *p* < 0.001, η_p_^2^ = 0.255) than those with angular geometries. For example, under all 100% high-load conditions, the curve space always maintains a higher pleasure score (M > 4.70) compared with angular spaces, while the average value of angular spaces is less than 4.00. This finding supports that streamlined forms significantly enhance the aesthetic preference (liking) and emotional valence (pleasure) of the space, while also fostering a greater sense of psychological control (dominance). These behavioral results align with evolutionary aesthetic theories (Bar & Neta, 2006; Reber et al., 2004), which posit an innate human preference for smooth contours, as they are generally perceived as more visually accommodating and less perceptually demanding than sharp, angular geometries.

Regarding color combination, a significant main effect has been observed. Compared with similar colors, contrasting colors significantly increased the degree of arousal (F (1, 60) = 4.09, *p* < 0.05, η_p_^2^ = 0.064) but negatively impacted pleasure (F (1, 60) = 6.73, *p* < 0.05, η_p_^2^ = 0.101) and liking (F (1, 60) = 13.39, *p* < 0.001, η_p_^2^ = 0.185). This evidence indicates that while high-contrast color schemes can strongly attract visual attention and elevate physiological arousal (Elliot, 2015), combining them with sharp, angular spatial geometries significantly increases the risk of cognitive overload, thereby reducing subjective aesthetic preference (Vartanian et al., 2013).

#### 3.1.2. Interactive Effects: Buffering and Amplification Effects

**Figure 5:**
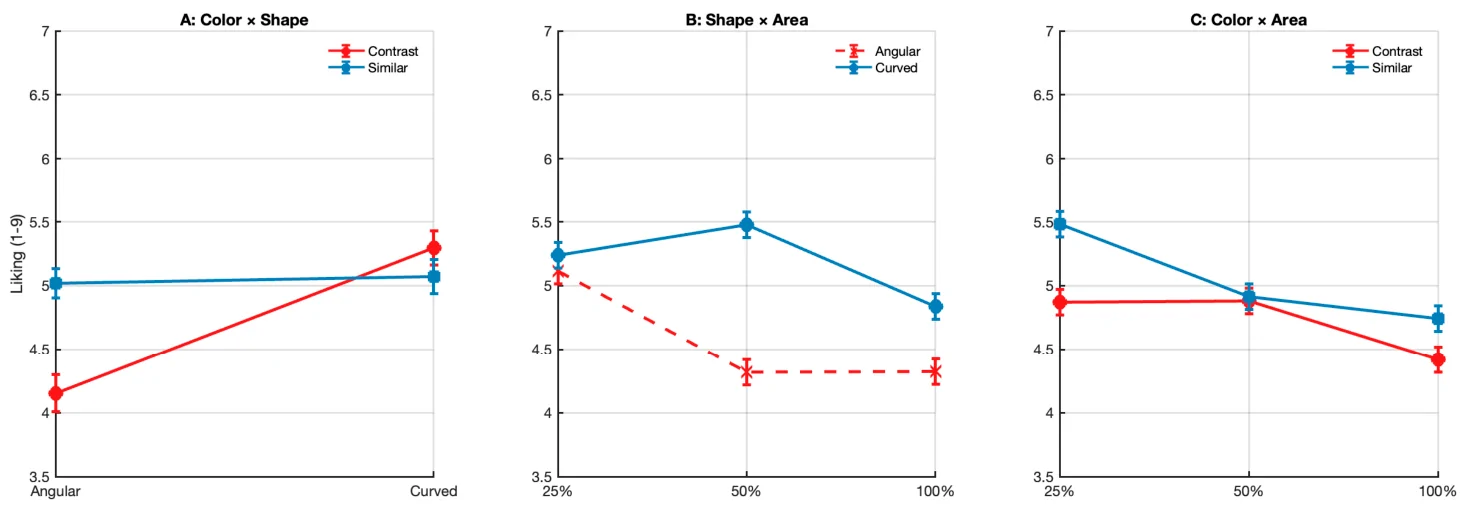
Two-way interaction effects of color, shape, and area proportion on subjective liking.

Beyond main effects, highly significant interactions revealed dynamic synergistic effects (Table 2). Specifically, the aesthetic buffer effect was visible in the Color × Shape interaction [F (1, 60) = 51.80, *p* < 0.001, η_p_^2^ = 0.46]. As shown in Figure 5A, the curve outline plays a positive buffering effect. Compared with the angular geometry, the Liking rating in the high-stress contrast color scene was significantly improved. Through the Shape × Area interaction [F (2, 120) = 16.47, *p* < 0.001, η_p_^2^ = 0.21], it was found that this morphological advantage has scale sensitivity: even if the environment expands to 100% of the spatial scale, the preference for curve geometry remains stable (Figure 5B). In addition, the quantitative amplifier effect is confirmed by the Color × Area interaction [F (2, 120) = 8.25, *p* < 0.001, η_p_^2^ = 0.12]. As shown in Figure 5C, the Area proportion significantly affects the pressure; for contrasting colors, the liking score drops significantly from 4.87 ± 0.12 at the 25% baseline to the significant emotional depression of 4.42 ± 0.14 at 100% coverage, which shows that the increase in spatial scale results in a significant amplification effect, making the negative perception of color conflict stronger.

**Figure 6:**
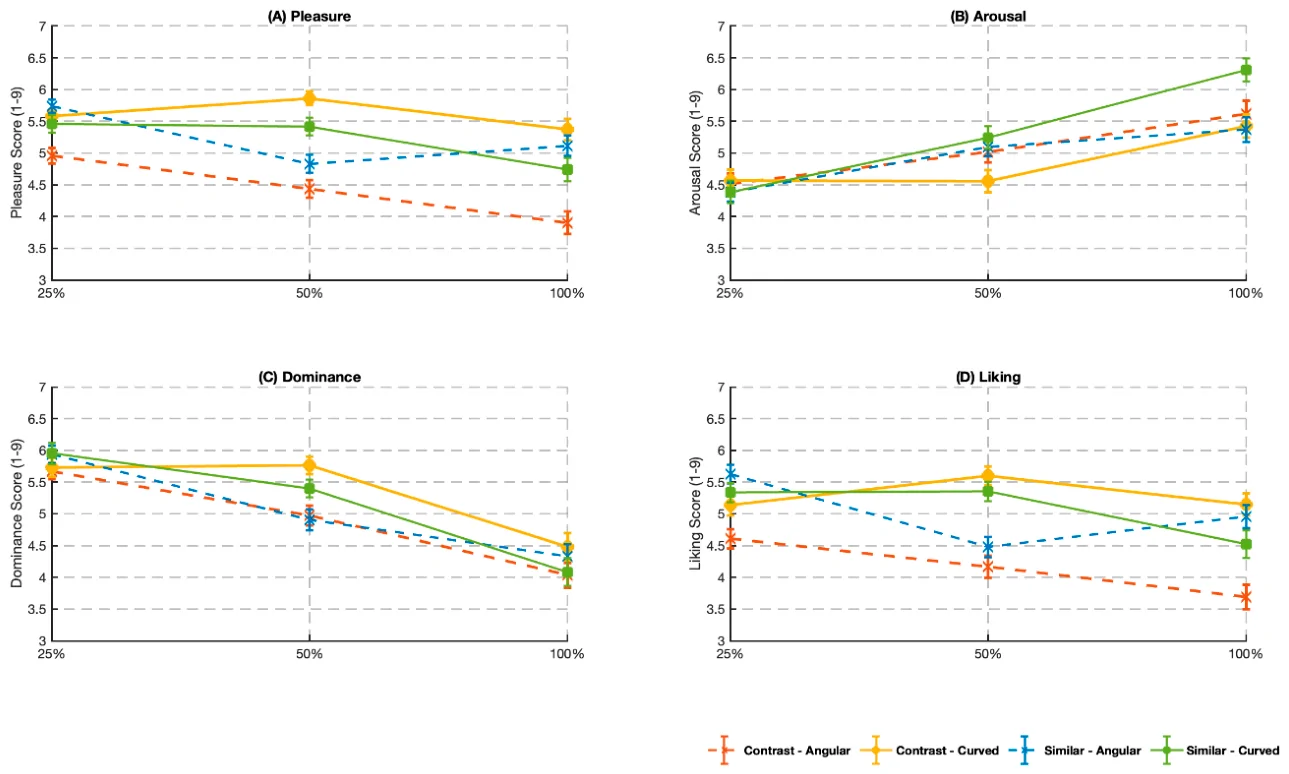
The three-way interactions of color, shape, and area proportion across all SAM dimensions (Pleasure, Arousal, Dominance, and Liking). Note. The error bars represent standard error of the mean (SEM).

As shown in the interaction diagram (Figure 6), with the expansion of the spatial scale, the progressive influence of form becomes crucial. At the baseline level of 25%, although the curve shape has shown a preliminary advantage in pleasure and liking, the overall emotional difference is relatively limited. However, when the high-contrast color scheme is extended to the extreme 100% coverage, there is an obvious aesthetic collapse under the angular condition (red dotted line). In this sensory overload area, the liking score plummeted to 3.69 ± 1.49 (Figure 6D), and the pleasure score also fell to the critical low of 3.90 (Figure 6A).

Crucially, the morphological buffering effect achieved by replacing the edge with the curve outline is very significant (orange solid line). Under the same 100% contrast condition, the curve intervention successfully pulled the liking score back to the stable 5.15 ± 1.38, and the pleasure and dominance also achieved a parallel recovery. Interestingly, the analysis of the arousal (Figure 6B) shows that the curve intervention not only slightly buffers the extreme physiological surge caused by the edge, but more importantly, it recalibrates the user’s high-arousal state by improving the pleasure through synchronization. This transforms the spatial experience from threat-related pain to aesthetic excitement, proving that curve boundaries act as a powerful cognitive buffer, transforming highly conflicting space into a perceptually balanced environment.

Behavior and subjective data present hierarchical emotional response spatial parameters. Area proportion and color combination are stressors that exacerbate psychological load, and the curve form is the key buffering effect for reverse aesthetic collapse in extreme settings. However, subjective evaluation is only related to the conscious part of aesthetic experience. To further clarify the neural effect behind it—specifically, whether this buffering occurs in the early automatic stage of sensory processing or in the later cognitive evaluation stage—the next section will analyze the neurophysiological response (EEG), focusing on the N200 component and the FAA.

### 3.2. Objective Neurophysiological Responses

The RM-ANOVA of neurophysiological indicators (Table 3) provided objective evidence of how spatial color parameters modulate early cognitive processing and motivational tendencies. Regarding the independent effect, the N200 component as a conflict detection and early attention sign shows the shape [F (1, 60) = 43.43, *p* < 001, η_p_^2^ = 0.42], color [F (1, 60) = 12.19, *p* = 0.001, η_p_^2^ = 0.17], and area [F (2, 120) = 5.75, *p* = 0.011, η_p_^2^ = 0.09]. All show significant main effects. Specifically, angular geometry and contrasting colors induce significantly greater negative N200 amplitude, indicating a higher degree of early neural conflict, reflecting processes such as conflict monitoring and mismatch detection. Furthermore, Frontal Alpha Asymmetry (FAA) was significantly modulated by color [F (1, 60) = 11.05, *p* = 0.002, η_p_^2^ = 0.16], and contrasting colors triggered a right prefrontal advantage, which indicates that the avoidance motivation is enhanced (Harmon-Jones et al., 2010). In addition to the independent effect, the significant “Color × Shape” interaction in the N200 [F (1, 60) = 9.81, *p* = 0.003, η_p_^2^ = 0.14] indicates the aesthetic buffering mechanism. This interaction shows that in the early stage, morphological contours have a profound impact on the neural processing of color conflict. Most importantly, the significant three-factor interaction for the N200 [F (2, 120) = 10.98, *p* < 0.001, η_p_^2^ = 0.16] fully depicts the buffering effect. As shown in the interaction diagram (Figure 6) and the scalp distribution diagram (Figure 6C), when paired with the angular shape, the neural conflict reaches its peak under the condition of 100% area + contrasting color. However, after replacing the angular shape with a curved outline, this negative N200 amplitude is significantly weakened. The lack of significant three-way interaction in late LPP and continuous Beta oscillation indicates that this buffering is an automatic, preconscious process that occurs during early sensory conflict detection, rather than an intentional reassessment in the late stage.

#### 3.2.1. Early Cognitive Conflict and the Attenuation of Visual Stress

**Figure 7:**
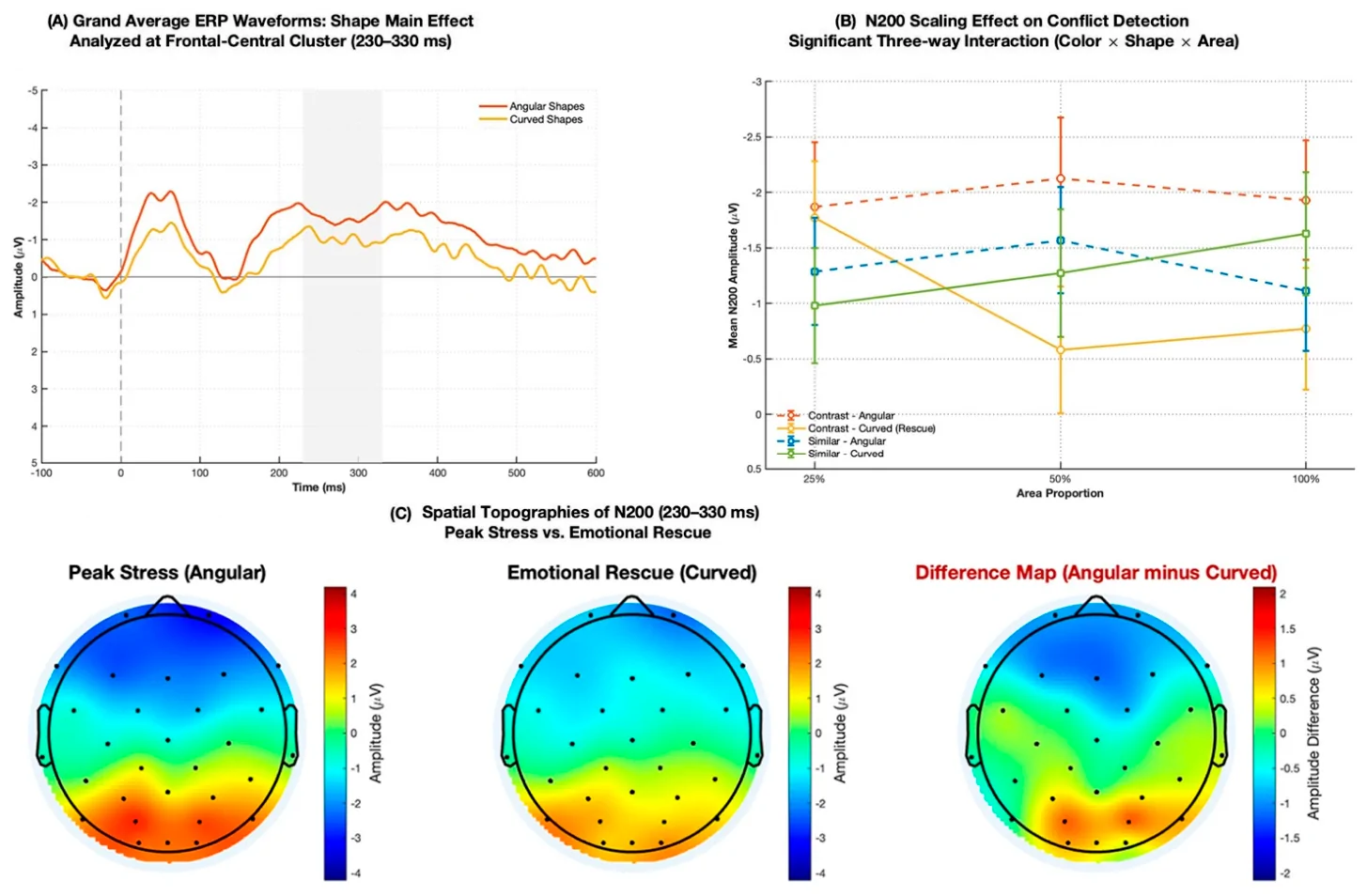
Objective neurophysiological evidence of the buffering effect (Note: In panels (**A**,**B**), negative amplitude is plotted upwards, adhering to standard electrophysiological conventions). The gray shaded area represents the time window (230–330 ms) used for the N200 statistical analysis.

Figure 7 shows the electrophysiological evidence of early cognitive conflict processing with the N200 component of the central region of the forehead (230–330 ms) as an indicator. As shown in the total average waveform diagram (Figure 7A), compared with the curve outline, the angular geometry induces a more significant negative N200 deflection. This observation aligns with the stable main effect of shape (F (1, 60) = 43.432, *p* < 0.001, η_p_^2^ = 0.42), indicating that sharp spatial features triggered a significantly larger negative amplitude (MD = −3.078 μV, 95% CI [−4.012, −2.143], Cohen’s d = 0.84), reflecting stronger and automatic frontal attention, conflict detection, and perception incongruence.

Critically, this early conflict processing is dynamically regulated by a hierarchical amplification effect, which is confirmed by the highly significant three-way interaction of color × shape × area (F (2, 120) = 10.981, *p* < 0.001, η_p_^2^ = 0.155). As systematically detailed in Supplementary Table S2, Mauchly’s test indicated that the assumption of sphericity was met for this specific three-way interaction; therefore, uncorrected degrees of freedom were used. For other neurophysiological effects where sphericity was violated, Greenhouse–Geisser corrections were applied accordingly. In the scaling effect diagram (Figure 7B), under the contrast–angular condition (orange dotted line), the amplitude of N200 gradually increases with the increase in the spatial scale, and reaches an extreme negative level at 100% coverage. On the contrary, under the same high-arousal contrast color condition, when the angular boundary is replaced by a curve geometry (yellow solid line), the amplitude of N200 is greatly attenuated, and its growth trend with the change in area becomes obviously flat. This shows that the curve morphology can effectively suppress the conflict amplification effect caused by high-intensity visual configuration.

The spatial topographic map (Figure 7C) further supports this buffer effect. The difference topography (angular minus curved) reveals a deep differential activation cluster on the frontal–central electrode. This not only represents a stronger overall activation, but also reflects the redistribution of neuroprocessing intensity, indicating that the curve geometry significantly reduces the input of neural resources associated with the conflict. In summary, these findings provide aggregated electrophysiological evidence for the early buffering effect: under high visual load conditions, the curve form, as a cognitive buffer in the pre-attentive stage, effectively weakened the conflict signal in about 250 ms. Ultimately, this highlights that spatial form is not only an aesthetic feature, but also a key functional factor for regulating neural stability in complex visual environments.

#### 3.2.2. Sustained Affective Evaluation and Cortical State

**Figure 8:**
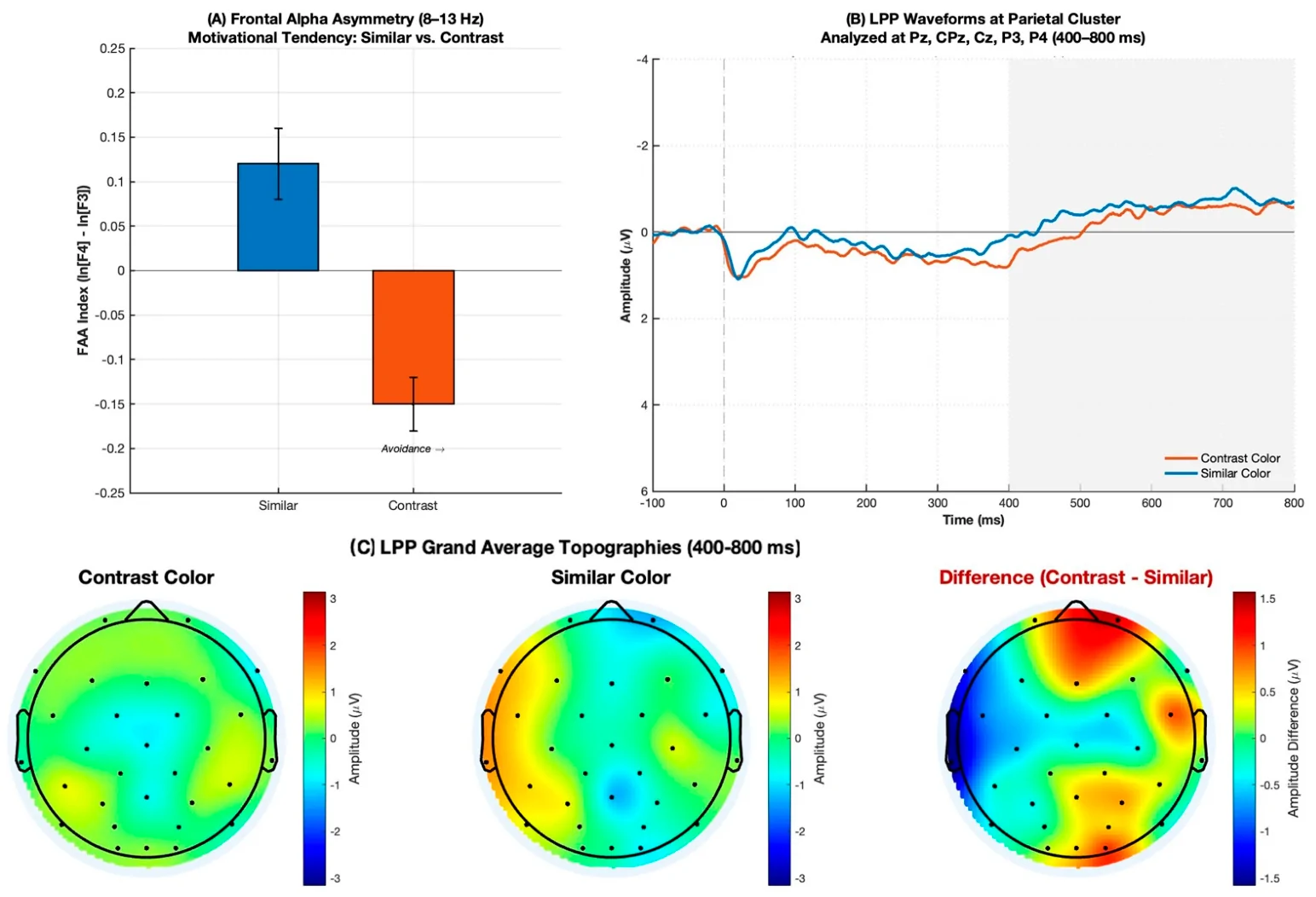
Sustained affective evaluation and cortical overload driven by color combinations. Blue and orange colors denote the Similar and Contrast color conditions, respectively. In panel (**A**), error bars repre-sent the standard error of the mean (SEM). In panel (**B**), the vertical dotted line at 0 ms indicates the stimulus onset, and the gray shaded area represents the time window (400–800 ms) used for the LPP statistical analysis.

As shown in Figure 8A, the RM-ANOVA found that color combinations had a very significant main effect on the FAA index (F (1, 60) = 11.053, *p* = 0.002, η_p_^2^ = 0.156). Compared with the positive approach-related tendency induced by similar colors (blue column), exposure to contrasting colors (orange column) leads to a significant negative offset in FAA (MD = −0.204, 95% CI [−0.326, −0.081], Cohen’s d = 0.43). Under the premise that FAA calculates according to the alpha power of the right minus the left, this negative offset reflects the relatively stronger activation of the right prefrontal cortex.

Concurrently, Color has a main effect on the LPP amplitude (F (1, 60) = 4.316, *p* = 0.042, η_p_^2^ = 0.067), indicating that color contrast strongly regulates continuous emotional processing. In the grand average waveforms at the parietal cluster (Figure 8B), the positive deflection induced by the high-arousal contrast color (orange line) in 400–800 ms is significantly greater than that of the similar color (blue line) (MD = 0.479 μV, 95% CI [0.018, 0.940], Cohen’s d = 0.27). The topographic map (Figure 8C) further indicates this, revealing that the significant positive difference (red area) is highly concentrated in the mid-parietal cortex, which is consistent with the typical spatial distribution profile of LPP. This model shows that the high-contrast configuration significantly enhances emotional salience and continuous attention resource allocation.

In summary, these findings reveal a clear time separation effect in spatial perception processing: shape mainly regulates early conflict detection (N200), while color contrast mainly drives late continuous arousal and motivation evaluation (LPP and FAA). Unlike passively reducing the impact of perception, the high-contrast environment prolonged neural engagement. They continued to occupy top-down attention resources to maintain the elevated level of arousal, thus promoting the biological system to favor the avoidance-oriented state of motivation.

### 3.3. Triangulation of Subjective and Objective Measures

**Figure 9:**
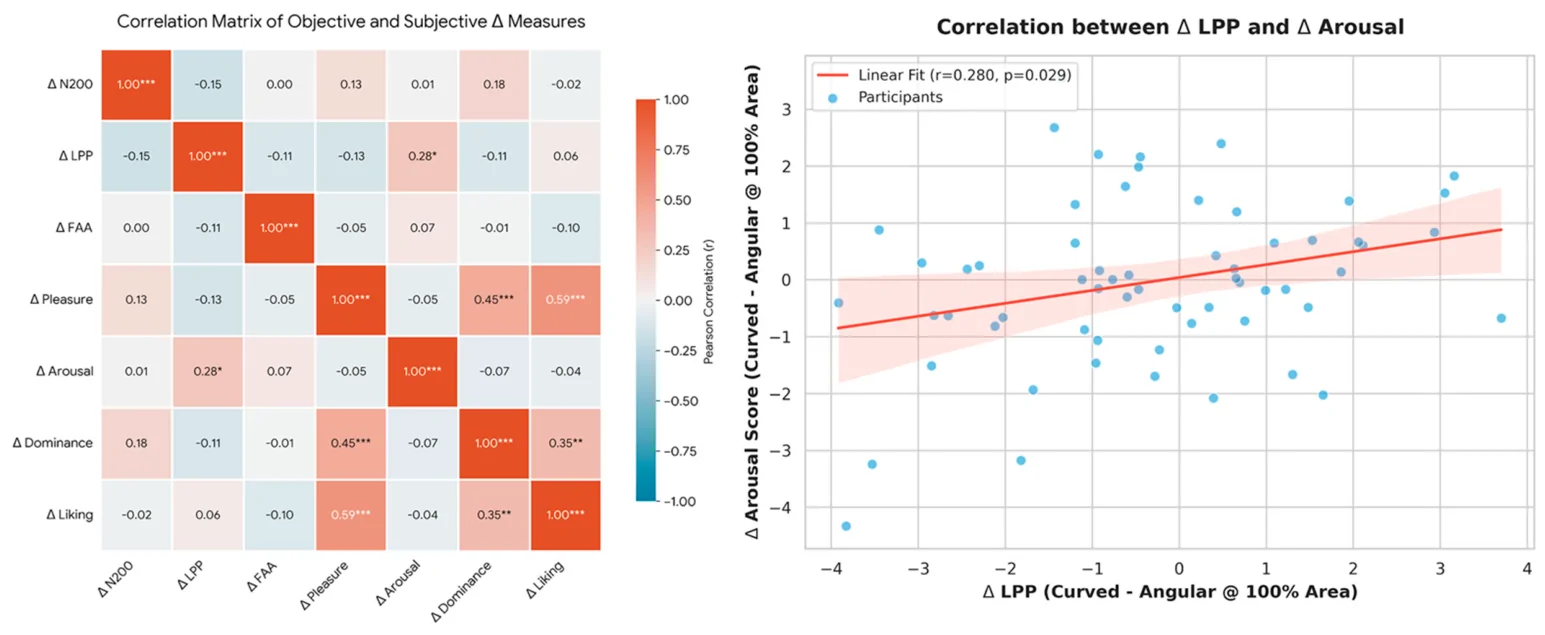
Correlation matrix for the triangulation of subjective and objective measures underlying the emotional buffering effect. * *p* < 0.05, ** *p* < 0.01, *** *p* < 0.001.

To verify the behavioral correlation of the buffering effect, this study uses Person correlation analysis to test the incremental change between neurophysiological markers and subjective evaluation (Δ: Curved minus Angular at the 100% spatial area condition). The correlation matrix (Figure 9) shows that an obvious separation was observed between the objective neurophysiological response and the conscious psychological state. While only a limited correlation was found, specifically a modest positive correlation between ΔLPP and Arousal (ΔArousal, r = 0.28, *p* = 0.029), this result suggests that the positive shift in LPP may be associated with the increase in subjective emotional arousal.

In contrast, the mappings between early cognitive markers (ΔN200, ΔFAA) and high-order aesthetic evaluations (ΔLiking, ΔPleasure) exhibited a functional dissociation (all *p* > 0.05). This discrepancy is theoretically revealing rather than contradictory; it may suggest that early neurophysiological responses and conscious aesthetic evaluations capture partially independent aspects of environmental perception. Specifically, N200 and FAA may reflect rapid affective responses occurring during early perceptual processing, whereas subjective evaluations (Liking and Pleasure) are likely subject to high-order cognitive appraisal and individual preference. Therefore, the lack of correlation does not “deny” our research results, but suggests that neurophysiological indicators may reveal implicit affective responses that are not fully reflected in conscious self-reports. These findings provide preliminary evidence consistent with the dual-system processing framework of architectural aesthetics (Chatterjee & Vartanian, 2014). These findings show that streamlined spatial forms can buffer visual stress at the neural level, even if these implicit neural benefits are not fully transformed into conscious subjective preferences.

## 4. Discussion

### 4.1. Hierarchical Processing and the Morphological Buffering Effect

The interactive buffering-interference effect (color × shape) is the core of dynamic coupling in early cognitive processing. Previous evolutionary aesthetics research has shown that distinct contours transmit threat information and activate the rapid defense pathway (Larson et al., 2009). Neuroimaging evidence shows that angular visual elements trigger high-level activation of the amygdala before conscious recognition, and the amygdala is very important for fear conditioning and threat detection (Bar & Neta, 2007). Our research results extend the theory to three-dimensional architectural spaces. Angular shapes elicited a larger N200 amplitude, which indicates that early conflict detection is stronger. From an evolutionary perspective, modern orthogonal architectural environments often present evolutionary mismatches. The human brain evolved in natural environments with organic, curved, and fractal geometric shapes. Consequently, the visual cortex processes sharp artificial edges with higher neural friction (Biederman & Vessel, 2006). It is worth noting that replacing angular shapes with curved contours significantly attenuated these conflict-related responses, and curved contours attenuated conflict-related neural responses under high-contrast conditions. These findings are consistent with Processing Fluency Theory and suggest that curved contours facilitate perceptual integration under visually demanding conditions in built environments (Reber et al., 2004). The human visual cortex expends less cognitive effort and has less neural friction when processing smooth curves compared to processing sharp angles. This view is strongly supported by the recent neuro-aesthetic framework (Chatterjee & Vartanian, 2014).

### 4.2. Area Proportion as a Quantitative Amplifier of Visual Stress

While previous assumptions often regarded spatial scale as an independent driver of specific emotions, our data reveal a more nuanced reality: although area proportion exhibits a main effect on early conflict detection (N200), its most critical regulatory function emerges in the profound three-way interaction on the N200 component, acting as a quantitative amplifier. The scale of space does not directly create specific emotions, but it significantly amplifies the perceptual deviations originally induced by color and shape configurations. This is in line with the environmental load theory, which points out that when the proportion of stimuli in a person’s field of vision is relatively large, especially in an immersive 3D environment (Banaei et al., 2017), it will greatly test the ability of the nervous system to process sensory input. According to the visual field and Prospect-Refuge theory, large-scale spaces (100%) with angular and high-contrast elements can extremely limit the visual refuge and will exceed the bandwidth of the senses (Stamps, 2005). Specifically, when the stress configuration (contrast + angle) expands to 100%, neural conflicts lead to aesthetic collapse.

### 4.3. Spatiotemporal Dynamics: Shape-Driven Valence Versus Color-Driven Arousal

This study makes use of the high temporal resolution of electroencephalogram (EEG) to distinguish the spatiotemporal roles of form and color in spatial perception and shows that there are different functions. Morphological features are mainly associated with the N200 and FAA and determine approach or avoidance motor orientation. Conversely, color combinations significantly affect the late emotional process, and high-contrast colors trigger stronger subjective arousal and higher late positive potential (LPP) amplitudes. The precise chronological order fundamentally reflects how the human brain evaluates the spatial environment for survival. Processing shape is a fundamental need to quickly identify physical boundaries, obstacles or immediate threats. And regarding color, the perception of a certain environmental energy and temperature requires more lasting semantic evaluation (Kveraga et al., 2007). According to the color-in-context theory (Elliot & Maier, 2014), high chromatic contrast in the natural environment often acts as a warning signal or an indicator of high environmental energy. So, the brain remaining alert and in a high-arousal state to monitor these color signals is an embodiment of biological adaptability. The observed temporal dissociation is broadly consistent with the motivated attention framework (Bradley, 2009). Spatial geometry encodes threat and safety, and color contrast makes perception more obvious, and the brain subsequently allocates attention resources (Hajcak et al., 2010).

### 4.4. Selective Inter-Modal Coupling: Arousal–Valence Asymmetry and Cognitive Over-Riding

The key contribution of this research is the discovery of selective cross-modal coupling that can be explained by Russell’s Circumplex Model of Emotion (Russell, 1980). Emotional experience is defined by two independent dimensions, namely arousal and valence. The results of this study show that there is a basic asymmetry when processing these dimensions in an architectural environment.

Coupling is evident in arousal: the increase in sustained cortical activation predicts the rise in participants’ subjective arousal. Conversely, there is a functional dissociation in terms of valence. Although curved shapes provide preconscious emotional buffering, these physiological benefits do not linearly translate into subjective liking or pleasure.

This aesthetic evaluation shown by such decoupling is consistent with a top-down high order cognitive structure. Therefore, the present findings should be interpreted as providing preliminary neurophysiological evidence consistent with a dual-system perspective, rather than a direct validation of Kahneman’s dual-process theory (Kahneman, 2011) in architectural experience. Furthermore, alternative explanations for this dissociation must be considered. First, EEG and SAM ratings capture responses across fundamentally different time scales; N200 reflects transient, pre-attentive sensory processing, whereas subjective liking is a retrospective evaluation. Second, the absence of a simple linear correlation does not preclude complex, nonlinear interactions between early neural conflict and ultimate preference. Finally, top-down contextual factors and individual aesthetic sensitivity may introduce substantial variance in self-reported data, potentially overshadowing the subtle baseline neural shifts. As suggested by the Model of Aesthetic Appreciation (Leder et al., 2004), forms may use biosafety signals to modulate the early sensory system (System 1: fast, automatic), but the final conscious judgment (System 2: prudent, explicit) is highly regulated by professional training, cultural background, and trait preferences. For example, environmental psychology research shows that there are persistent aesthetic differences between well-trained architects and ordinary people; architects, due to professional training, often give relatively high subjective scores to brutalist or concrete structures with more edges and corners, surpassing the innate biosafety threat response of System 1 (Gifford et al., 2000).

### 4.5. Practical Implications for Evidence-Based Architectural Design

It is crucial to emphasize that the following recommendations are derived from controlled virtual simulations and should strictly be framed as preliminary insights rather than definitive evidence-based design guidelines. These suggestions require further validation in complex, real-world architectural settings with diverse multisensory conditions.

First, the curved geometric shape could potentially act as a form buffering factor to alleviate the high arousal and visual stress induced by high-contrast color schemes. This emotional buffering might be beneficial in high-stress architectural environments such as healthcare or intensive workspaces. These initial guidelines serve as a theoretical basis for future architectural design, suggesting that designers might consider smooth curved boundaries to support the visual nervous system while not compromising functional efficiency (Ulrich et al., 2008). According to Attention Restoration Theory (ART) (Kaplan, 1995), continuous high-load visual stimulation consumes directed attention, thereby leading to cognitive fatigue. Designers may use curves to create restorative micro-environments, which can potentially reduce early N200 conflicts under specific visual configurations, and passively supplement cognitive resources. This morphological buffering becomes particularly critical when high-contrast elements occupy moderate visual fields, such as the 50% area proportion tested in our study. Secondly, spatial scale may amplify perceptual biases, and designers need to be cautious when using large-area high-contrast color palettes—especially at the fully enveloping 100% proportion—to avoid cognitive overload. Conversely, at a localized 25% proportion, high-contrast or angular elements can be more safely applied as dynamic focal points without causing excessive visual strain. Finally, designers should maintain the consistency of form and color emotions, such as matching smooth curves with harmonious color schemes, to improve perceptual comfort and approach behavior, and to optimize the well-being of users in immersive spaces. This approach enables architects to make design decisions based on objective neural health conditions rather than just subjective intuition.

### 4.6. Limitations and Future Directions

This study provides powerful neurophysiological understanding of indoor color perception, but some limitations and directions for future research need to be noted.

First, ERP uses short exposures to capture acute neural dynamics, whereas long exposure situations exist in reality. Future longitudinal research needs to explore whether the emotional buffering of curved shapes can persist over hours or days, or whether perceptual habituation will weaken its buffering role.

Second, immersive virtual reality has a relatively high visual realism and can strictly control the experience, but it lacks the multisensory complexity of the physical environment. Subsequent research can apply mobile EEG systems in actual architectural environments to verify the research findings under ecologically complex conditions, and explore potential cross-modal sensory interactions.

Third, this study recorded neuro-emotional responses from a static view. However, human–building interaction is dynamic. Future paradigms should incorporate active navigation to evaluate how the optic flow of curved boundaries modulates continuous emotions and spatial memory.

Finally, most of the participants were college students. Although this sample provides a controlled baseline for neuro-aesthetic responses, it also limits the universal applicability of our research results. Aesthetic evaluation is often influenced by training and culture, and plays a role when top-down cognition dominates. For example, professional architectural training may cultivate aesthetic expertise, so that experts no longer regard sharp angle geometry as a threat, but as a structurally interesting point, which may inhibit the instinctive safety response of System 1. Similarly, the decline in sensory processing ability caused by age, or spatial perception differences in different cultural backgrounds, may significantly change people’s emotional response. Future research needs to recruit diverse groups, including professional architects, the elderly, and individuals from different cultural backgrounds, to explore how professional experience and sociocultural factors regulate the interaction between implicit neural buffers and explicit aesthetic preferences. Moreover, the limited correspondence between neural indicators and subjective ratings highlights the complexity of translating implicit neurophysiological responses into conscious aesthetic judgments. Future studies combining neurophysiological measures with behavioral and longitudinal assessments are needed to further clarify this relationship.

## 5. Conclusions

The core scientific findings are summarized as follows:

Morphological Buffer Mecha: Color Spatial perception is not a simple addition of features, but a highly interactive process. Under extremely high-contrast color conditions, curved geometric figures were associated with reduced early neural conflict, greatly neutralizing early visual stress and cognitive conflicts.

Spatiotemporal Dissociation of Visual Features: Visual features have different spatiotemporal roles. Morphological features (shapes) dominate the early stage and determine approach–avoidance motivation (FAA). Conversely, color combinations drive the late stage and determine continuous environmental alertness (LPP).

Spatial Scale as a quantitative modulator: Area proportion does not function as an independent emotion generator but exerts a significant amplification effect. It exacerbates the pre-existing neuro-affective biases induced by color shape configurations, particularly intensifying stress responses under high-arousal conditions until they reach an aesthetic collapse.

Selective multi-modal coupling and dual-system framework: Robust cross-modal correlations between LPP and subjective arousal present bottom-up processing of environmental intensity. Functional dissociation between N200, FAA, and preference provides preliminary evidence consistent with dual-system architecture. Theoretically, the curved form may act as a preconscious safety baseline indicator, while the final aesthetic appreciation is an individual, top-down construction.

Ultimately, by bridging the gap between implicit neural processing and explicit spatial aesthetics, this research underscores the profound impact of our physical surroundings on mental health. Recognizing and applying these spatiotemporal neural dynamics will empower future architects to transition from subjective intuition to objective neuro-aesthetics, enabling the conscious design of habitats that not only mitigate cognitive fatigue but actively foster human psychological resilience and well-being.

## Figures and Tables

**Table 1:** Descriptive statistics for Pleasure, Arousal, Dominance, and Liking scores across experimental conditions (*N* = 61).

Color	Shape	Area	Pleasure	Arousal	Dominance	Liking
Contrast	Angular	25%	4.96 ± 0.95	4.52 ± 1.27	5.67 ± 0.97	4.61 ± 1.21
		50%	4.43 ± 1.10	5.02 ± 1.27	4.98 ± 1.21	4.16 ± 1.35
		100%	3.90 ± 1.40	5.62 ± 1.62	4.03 ± 1.53	3.69 ± 1.49
	Curved	25%	5.58 ± 0.81	4.57 ± 1.39	5.73 ± 1.17	5.14 ± 1.26
		50%	5.86 ± 0.85	4.56 ± 1.34	5.76 ± 1.09	5.60 ± 1.16
		100%	5.37 ± 1.29	5.43 ± 1.45	4.48 ± 1.68	5.15 ± 1.38
Similar	Angular	25%	5.74 ± 0.84	4.39 ± 1.15	5.93 ± 1.09	5.62 ± 1.15
		50%	4.83 ± 1.11	5.10 ± 1.18	4.90 ± 1.30	4.48 ± 1.25
		100%	5.12 ± 1.26	5.37 ± 1.50	4.33 ± 1.52	4.96 ± 1.42
	Curved	25%	5.46 ± 1.09	4.39 ± 1.39	5.95 ± 1.32	5.34 ± 1.20
		50%	5.42 ± 1.10	5.25 ± 1.41	5.40 ± 1.08	5.35 ± 1.21
		100%	4.74 ± 1.43	6.31 ± 1.43	4.08 ± 1.72	4.53 ± 1.68

Note. Values are presented as mean ± standard deviation (SD). All items were rated on a 9-point Likert scale.

**Table 2:** Summary of repeated measures ANOVA results for Pleasure, Arousal, Dominance, and Liking (*N* = 61).

Source	df	Pleasure	Arousal	Dominance	Liking
Main Effects					
Color	1, 60	6.73 * (*p* = 0.012, η_p_^2^ = 0.101)	4.09 * (*p* = 0.038, η_p_^2^ = 0.064)	0.02 (*p* = 0.603, η_p_^2^ = 0.003)	13.39 *** (*p* < 0.001, η_p_^2^ = 0.185)
Shape	1, 60	27.62 *** (*p* < 0.001, η_p_^2^ = 0.315)	0.64 (*p* = 0.27, η_p_^2^ = 0.011)	7.62 ** (*p* = 0.008, η_p_^2^ = 0.113)	20.58 *** (*p* < 0.001, η_p_^2^ = 0.255)
Area	2, 120	21.93 *** (*p* < 0.001, η_p_^2^ = 0.268)	75.99 *** (*p* < 0.001, η_p_^2^ = 0.559)	59.08 *** (*p* < 0.001, η_p_^2^ = 0.496)	10.88 *** (*p* < 0.001, η_p_^2^ = 0.158)
2-Way Interactions					
Color × Shape	1, 60	60.78 *** (*p* < 0.001, η_p_^2^ = 0.503)	13.27 *** (*p* < 0.001, η_p_^2^ = 0.181)	5.25 * (*p* = 0.026, η_p_^2^ = 0.080)	51.80 *** (*p* < 001, η_p_^2^ = 0.463)
Color × Area	2, 120	4.51 * (*p* = 0.013, η_p_^2^ = 0.07)	9.42 *** (*p* < 0.001, η_p_^2^ = 0.136)	3.48 * (*p* = 0.043, η_p_^2^ = 0.055)	8.25 *** (*p* < 0.001, η_p_^2^ = 0.121)
Shape × Area	2, 120	19.68 *** (*p* < 0.001, η_p_^2^ = 0.247)	5.92 ** (*p* = 0.004, η_p_^2^ = 0.09)	7.83 *** (*p* = 0.001, η_p_^2^ = 0.115)	16.47 *** (*p* < 0.001, η_p_^2^ = 0.215)
3-Way Interaction					
C × S × A	2, 120	11.56 *** (*p* < 0.001, η_p_^2^ = 0.163)	10.53 *** (*p* < 0.001, η_p_^2^= 0.149)	3.06 ^†^ (*p* = 0.051, η_p_^2^ = 0.048)	14.43 * (*p* < 0.01, η_p_^2^ = 0.194)

Note. The data presented in the table are F’ values. C = Color, S = Shape, A = Area. * *p* < 0.05, ** *p* < 0.01, *** *p* < 0.001, ^†^ indicates marginal significance. Degrees of freedom are presented in this table. Where the assumption of sphericity was violated, repeated measures ANOVAs were evaluated using corrected degrees of freedom (Greenhouse–Geisser or Huynh–Feldt estimates); please refer to Supplementary Table S2 for the exact corrected df values and epsilon estimates.

**Table 3:** Key RM-ANOVA results for neurophysiological indices.

Source	Metric	df	F	*p*	η_p_^2^
Main Effects					
Color (C)	N200	1, 60	12.189	0.001	0.169
	LPP	1, 60	4.316	0.042	0.067
	FAA	1, 60	11.053	0.002	0.156
Shape (S)	N200	1, 60	43.432	<0.001	0.42
Area (A)	N200	2, 120	5.753	0.011	0.087
Interactions (N200 focus)					
C × S	N200	1, 60	9.805	0.003	0.14
C × A	N200	2, 120	3.03	0.065	0.048
C × S × A	N200	2, 120	10.981	<0.001	0.155

Notes: For brevity, non-significant main effects of Shape and Area on LPP/FAA and all results for Beta oscillations are omitted (*p* > 0.05). Two-way and three-way interactions for LPP and FAA are also omitted as they did not reach statistical significance.

## Data Availability

The datasets generated and/or analyzed during the current study are available in the Mendeley Data repository, https://doi.org/10.17632/bxkgxg3ng4.1.

## Supplementary Materials

The following supporting information can be downloaded at https://www.mdpi.com/article/10.3390/bs16081364/s1, Table S1. Quantitative parameters of the color stimuli used in the experiment (sRGB space); Table S2. Summary of Mauchly’s test of sphericity and epsilon (ε) corrections for subjective and objective measurements.
