# Supplementary material for: Morphological Buffering in Neuro-Architecture: Interactive Effects of Color, Shape, and Area Proportion on Affective Processing
Source: Behav Sci (Basel). 2026 Aug 9;16(8):1364. doi: 10.3390/bs16081364 (PMC13509317; doi:10.3390/bs16081364)
Supplement: Supplementary file 1 [file behavsci-16-01364-s001.zip › behavsci-4397899-Table S1.pdf]

Table S1. Quantitative parameters of the color stimuli used in the experiment (sRGB space).

**Color Name**	**RGB (0-255)**	**HSV (H, S, V)**
Red	[255, 0, 0]	[0°, 100%, 100%]
Green	[0, 255, 0]	[120°, 100%, 100%]
Yellow	[255, 255, 0]	[60°, 100%, 100%]
Purple	[255, 0, 255]	[300°, 100%, 100%]
Orange	[255, 102, 0]	[24°, 100%, 100%]
Blue	[0, 0, 255]	[240°, 100%, 100%]

Note: Values reflect the material albedo settings in the Unity engine under Linear color space.
