# Supplementary material for: Morphological Buffering in Neuro-Architecture: Interactive Effects of Color, Shape, and Area Proportion on Affective Processing
Source: Behav Sci (Basel). 2026 Aug 9;16(8):1364. doi: 10.3390/bs16081364 (PMC13509317; doi:10.3390/bs16081364)
Supplement: Supplementary file 1 [file behavsci-16-01364-s001.zip › behavsci-4397899-Table S2.pdf]

Table S2. Summary of Mauchly's test of sphericity and epsilon (ε) corrections for subjective and objective measurements.

Dependent Variable	Effect	Mauchly's W	Approx. χ2	df	p-value	Greenhouse-Geisser ε	Huynh-Feldt ε
**Subjective Measure**							
Liking	Area Proportion	0.621	28.152	2	**< 0.001**	0.725	0.738
	Color × Area	0.937	3.823	2	0.148	0.941	0.97
	Shape × Area	0.958	2.523	2	0.283	0.96	0.991
	Color × Shape × Area	0.91	5.596	2	0.061	0.917	0.945
Arousal	Area Proportion	0.59	31.111	2	**< 0.001**	0.709	0.722
	Color × Area	0.971	1.765	2	0.414	0.971	1
	Shape × Area	0.952	2.9	2	0.235	0.954	0.985
	Color × Shape × Area	0.94	3.678	2	0.159	0.943	0.973
Dominance	Area Proportion	0.573	32.805	2	**< 0.001**	0.701	0.713
	Color × Area	0.806	12.744	2	**0.002**	0.837	0.859
	Shape × Area	0.918	5.059	2	0.08	0.924	0.952
	Color × Shape × Area	0.997	0.185	2	0.912	0.997	1
Valence	Area Proportion	0.67	23.635	2	**< 0.001**	0.752	0.767
	Color × Area	0.996	0.228	2	0.892	0.996	1
	Shape × Area	0.969	1.877	2	0.391	0.97	1

	Color × Shape × Area	1	0.022	2	0.989	1	1
**Objective (EEG) Measure**							
N200	Area Proportion	0.968	1.914	2	0.384	0.969	1
	Color × Area	0.996	0.218	2	0.897	0.996	1
	Shape × Area	0.903	6.046	2	**0.049**	0.911	0.938
	Color × Shape × Area	0.906	5.793	2	0.055	0.914	0.942
FAA	Area Proportion	0.981	1.112	2	0.573	0.982	1
	Color × Area	0.935	3.944	2	0.139	0.939	0.969
	Shape × Area	0.995	0.277	2	0.871	0.995	1
	Color × Shape × Area	0.935	3.959	2	0.138	0.939	0.968

Notes: Main effects of Color and Shape, as well as the Color × Shape interaction, are excluded from this table as they contain only two levels (df = 1) and therefore inherently satisfy the sphericity assumption. Statistical significance for Mauchly's test is evaluated at $\alpha = 0.05$. Where the sphericity assumption was violated ($p < 0.05$), degrees of freedom for the repeated-measures ANOVA were corrected. Following standard statistical guidelines, the Greenhouse-Geisser correction was applied when $\varepsilon < 0.75$, and the Huynh-Feldt correction was applied when $\varepsilon > 0.75$. Significant p-values indicating a violation of sphericity are highlighted in bold.
